# Accuracy assessment methods for physiological model selection toward evaluation of closed-loop controlled medical devices

**DOI:** 10.1371/journal.pone.0251001

**Published:** 2021-04-30

**Authors:** Ramin Bighamian, Jin-Oh Hahn, George Kramer, Christopher Scully

**Affiliations:** 1 Office of Science and Engineering Laboratories, Center for Devices and Radiological Health, United States Food and Drug Administration, Silver Spring, MD, United States of America; 2 Department of Mechanical Engineering, University of Maryland, College Park, MD, United States of America; 3 Department of Anesthesiology, The University of Texas Medical Branch, Galveston, TX, United States of America; Fuzhou University, CHINA

## Abstract

Physiological closed-loop controlled (PCLC) medical devices are complex systems integrating one or more medical devices with a patient’s physiology through closed-loop control algorithms; introducing many failure modes and parameters that impact performance. These control algorithms should be tested through safety and efficacy trials to compare their performance to the standard of care and determine whether there is sufficient evidence of safety for their use in real care setting. With this aim, credible mathematical models have been constructed and used throughout the development and evaluation phases of a PCLC medical device to support the engineering design and improve safety aspects. Uncertainties about the fidelity of these models and ambiguities about the choice of measures for modeling performance need to be addressed before a reliable PCLC evaluation can be achieved. This research develops tools for evaluating the accuracy of physiological models and establishes fundamental measures for predictive capability assessment across different physiological models. As a case study, we built a refined physiological model of blood volume (BV) response by expanding an original model we developed in our prior work. Using experimental data collected from 16 sheep undergoing hemorrhage and fluid resuscitation, first, we compared the calibration performance of the two candidate physiological models, i.e., original and refined, using root-mean-squared error (RMSE), Akiake information criterion (AIC), and a new multi-dimensional approach utilizing normalized features extracted from the fitting error. Compared to the original model, the refined model demonstrated a significant improvement in calibration performance in terms of RMSE (9%, *P* = 0.03) and multi-dimensional measure (48%, *P* = 0.02), while a comparable AIC between the two models verified that the enhanced calibration performance in the refined model is not due to data over-fitting. Second, we compared the physiological predictive capability of the two models under three different scenarios: prediction of subject-specific steady-state BV response, subject-specific transient BV response to hemorrhage perturbation, and leave-one-out inter-subject BV response. Results indicated enhanced accuracy and predictive capability for the refined physiological model with significantly larger proportion of measurements that were within the prediction envelope in the transient and leave-one-out prediction scenarios (*P* < 0.02). All together, this study helps to identify and merge new methods for credibility assessment and physiological model selection, leading to a more efficient process for PCLC medical device evaluation.

## Introduction

Physiological closed-loop controlled (PCLC) medical devices pertain to a rapidly advancing technology that work based on feedback from physiological sensors to make their own decisions for patient treatment without human input. These devices and their algorithms have been studied across a range of different applications [1–7]. During the 2015 public workshop on PCLC challenges and opportunities held by the U.S. Food and Drug Administration (FDA), it was emphasized that compared to the standard of care, a well-designed PCLC medical device can benefit patients, physicians, and more broadly the quality of healthcare by ensuring the delivery of effective therapy with reduced side effects and length of hospital stay and lowering the level of workload in hospitals [8, 9]. These potential benefits have inspired researchers to introduce automation in the biomedical instruments ever since, to which the Transparency Market Research has predicted a compound annual growth rate of 8.8% for the global healthcare automation market over the forecast period of 2017-2025 [10].

The autonomous nature of PCLC medical devices, however, has made researchers approach with a certain degree of caution. The decision for dose adjustment and treatment delivery made by the technology instead of human clinicians introduces new sources of hazard and safety issues for patients, such as algorithm robustness, stability, and hardware-software integrability [9, 11, 12]. Furthermore, the automation brings complexity to the design and use of the biomedical devices as compared to their use in manual care [8, 9]. Due to these challenges, development of tools and verification methods for the safety properties of PCLC medical devices is essential before they can be used in practice.

To take advantage of the technology’s promise against the given potential drawbacks inherent in the use of a PCLC medical device, appropriate hardware and software design as well as evaluation techniques must be ensured to demonstrate the performance and maximize safety precautions required before the device becomes available to patients. This can be obtained by a combination of data from nonclinical laboratory, animal, and clinical testing [13, 14]. Laboratory studies involve controlled environments that are different from those of standard care, while animal studies and clinical trials are expensive in time, effort and costs and have to comply with ethical requirements; thus, are often limited and prohibitive. For a specific intended use, running a small number of clinical studies does not guarantee that a device is safe everywhere, particularly for automated PCLC medical devices that may have performance affected by various patient responses and disturbance conditions that may not occur during a clinical study [15]. Due to these challenges, computational modeling has been proposed as an alternative approach to answer the critical questions related to the safety and effectiveness of a device [14]. Well-designed mathematical models can generate physiological variables corresponding to virtual patients and satisfy the need for creating inter- and intra-subject variability. They can be used to simulate wide spectrum of patient physiology and possibly their interaction with sensors and actuators, including those associated with the worst-case conditions that are less likely to happen in a limited number of clinical trials.

Uncertainties about the quality and fidelity of mathematical models and ambiguities about choice of measures for modeling performance need to be addressed before PCLC medical devices can be reliably evaluated. This can be done through validation processes to quantitatively establish the extent to which a mathematical model is an adequate representation of the real world [16]. Validation process in the field of medical device is an open-ended task and includes response assessment of mathematical models with respect to the real physiology. These models are often complex, with diverse structure and strong coupling between physiological compartments and mechanisms. Due to these complexities, conceptual questions immediately arise regarding proper ways to assess the adequacy of such mathematical models. In particular, the accuracy of the models may not be evaluated in a substantive way, except when inputs, boundary conditions, and model predictions are in very close vicinity of conditions in the calibration database [17]. Prior studies have mostly quantified the accuracy by comparing experimental data and responses computed by a mathematical model calibrated (fitted) to those data, assuming that the validation domain and its intended use fall within the experimental inputs and conditions used for model calibration (e.g., [18–20]). For a model to be used for PCLC medical device assessment, however, inputs and boundary conditions could be outside of the ones in calibration data. Thus, a mathematical model should be tested in terms of its predictive capability against physiological states and conditions for which it has not been calibrated, via numerical interpolation or extrapolation of the model to specific conditions defined by its intended use [21].

While mathematical models have been proposed in different physiological domains, there is no consensus about criteria for proper model selection toward evaluating PCLC medical devices. In particular, no guidance is available on proper number of parameters, goodness of fit, or predictive capability of a mathematical model for an effective PCLC medical device assessment. Furthermore, the mathematical model must not be judged based on the PCLC testing results since the PCLC medical device performance does not indicate the extent to which the mathematical model adequately represents physiological measurements—thus, assessment of a computational model should be performed in advance and independently from the PCLC medical device evaluation. Instead, a comparison can be made between different physiological models to choose the one with the best calibration performance while not overfitting the data due to a large number of parameters, and the best predictive capability during different treatment scenarios. Hence, it is essential to establish methods to assess both calibration performances and predictive capability across multiple candidate physiological models for PCLC medical device assessment.

To address the above mentioned challenge, this research develops tools for properly assessing accuracy of physiological models and establishes fundamental measures for evaluating predictive capability across different mathematical models. This research only engages in comparing the physiology-based first principles models —which as opposed to their black-box counterparts, are limited in their variety due to the use of physiological principles. Therefore, as a case study, we examine the adequacy of lumped-parameter mathematical models of patient physiology developed for evaluating PCLC fluid resuscitation devices. We build a refined physiological model of blood volume (BV) response by expanding an original model we developed in our prior research [19, 20]. We use the experimental data collected from sheep subjects undergoing hemorrhage and fluid resuscitation. First, we compare the calibration performance of two candidate physiological models, i.e., original and refined, using root-mean-squared error (RMSE), Akiake information criterion (AIC), and a new multi-dimensional approach that utilizes normalized features extracted from fitting error. Then, the accuracy and predictive capability of the two mathematical models are compared under three different scenarios, i.e., their use in 1) subject-specific prediction of steady-state response, 2) subject-specific interpolation of transient response to hemorrhage, and 3) prediction of the entire (transient and steady-state) response through a leave-one-out procedure. While this research proposes tools and methods for selecting the best between multiple candidate physiological models, it does not intend to provide recommendations on whether or not further refinement is needed for a mathematical model before it can be used for the PCLC medical device evaluation.

The remainder of this paper is organized as follows: section Materials and Methods presents two mathematical models of BV response to hemorrhage and fluid resuscitation that are compared in this research, describes the animal data used, and elaborates on the tools employed for model accuracy and predictive capability assessment. The comparison between the two candidates mathematical models is then made under three different scenarios, which is presented and discussed in Results and Discussion sections.

## Materials and methods

In this section, we first present the two candidate physiological models and elaborate how the original model is expanded to develop the refined model. After describing the experimental data, we present the parameter identifiability analysis for each model, parameter estimation procedures, and the methods employed for model calibration assessment and evaluation of the model accuracy and predictive capability between the two candidate models.

### Candidate physiological models of blood volume response

In this section, we briefly present the original model of BV response. Next, we show how this model is expanded to develop the refined model of BV response.

#### Model 1: The original BV model

**Fig 1:**
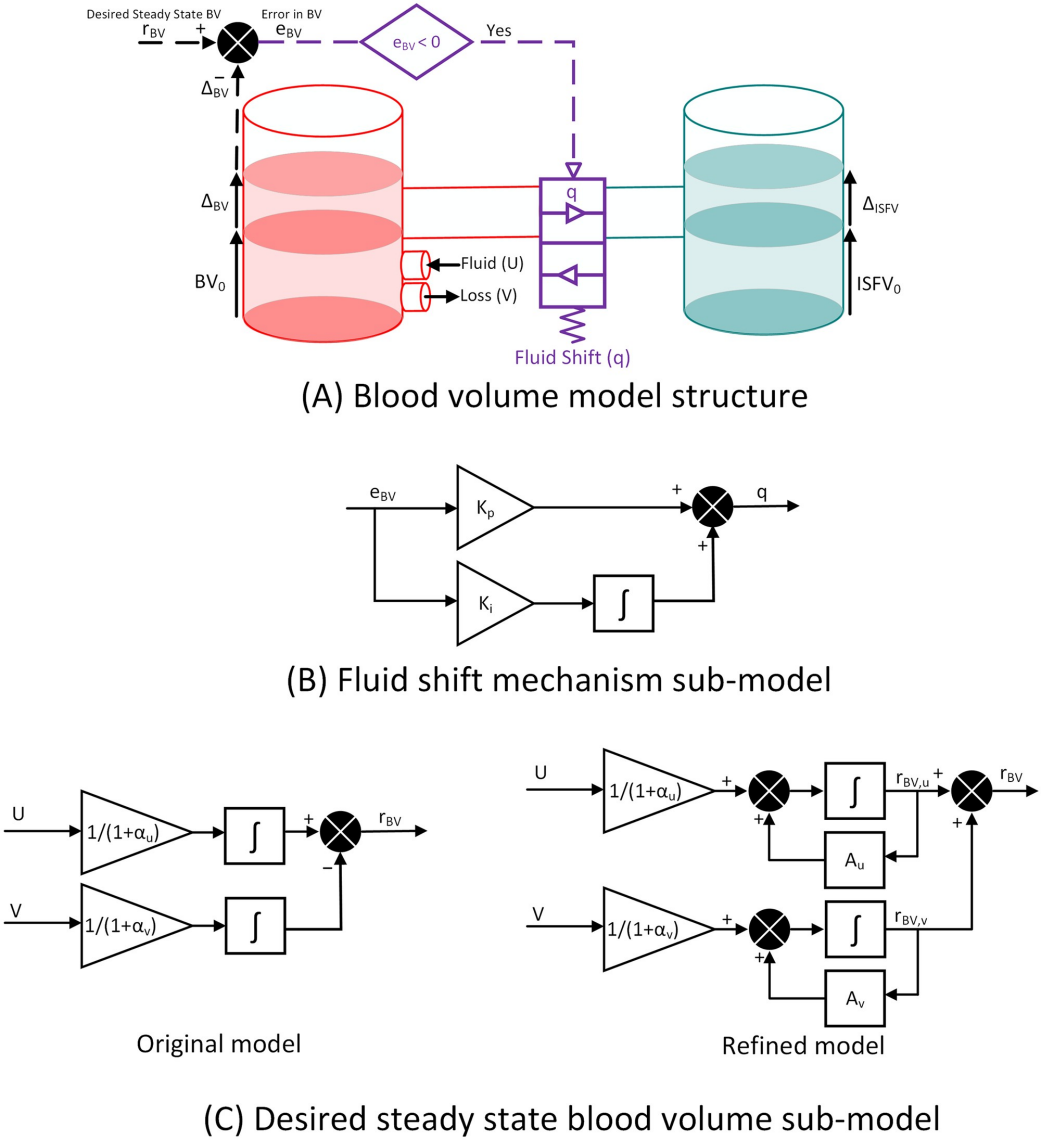
Lumped parameter model of blood volume (BV) response to fluid infusion and hemorrhage. (A): The model includes intravascular and interstitial (extravascular) fluid compartments with a bi-directional inter-compartmental fluid shift. The sign of *e*_*BV*_, which is the discrepancy between the change in BV (Δ*BV*) and desired steady-state change in BV (*r*_*BV*_), determines the direction of shift. As shown in the figure, a negative *e*_*BV*_ sets a shift of fluid toward interstitial compartment. (B): Fluid shift is modeled through a control-theoretic approach (see part A) and computed through the proportional-integral response to *e*_*BV*_; a larger *e*_*BV*_ leads to a larger amount of shift to achieve desired response. (C): *r*_*BV*_ is shown for the original and refined model. This is the main difference between the two models, in which *r*_*BV*_ in the refined model is affected by both the rate of infusion and loss and the current desired state of BV response for infusion and loss (right picture), while *r*_*BV*_ in the original model is only determined based on the rate of infusion and loss (left picture).

The details on this model can be found in our previous research [19, 20]. This mathematical model is built based on the physiological principle related to body fluid balance, indicating a gain (fluid infusion) or loss (hemorrhage and urinary output) perturbation in BV is distributed between the intravascular and interstitial fluid compartments to regulate the ratio between their volume changes [22] (see Fig 1). This distribution mechanism is modeled through a feedback controller that adjusts the rate of fluid shift between the two compartments to achieve a desired steady-state BV response (see Fig 1A and 1B). Since the inter-compartmental fluid shift behaves differently under the gain or loss situation due to the different compositions of the involved fluids, the model assumes distinct distribution ratios for infusion versus loss rate (see Fig 1C, left picture) [20]. For a given rate of infusion *U* and loss *V* at each time *t*, the desired steady-state change in BV, *r*_*BV*_, is defined as:

$$\begin{array}{c} r_{BV}\left(t\right)=\frac{1}{1+\alpha_{u}}\int_{0}^{t}U\left(\tau \right)d\tau -\frac{1}{1+\alpha_{v}}\int_{0}^{t}V\left(\tau \right)d\tau \end{array}$$ (1)

where *α*_*u*_ and *α*_*v*_ denote the fluid distribution ratio between intravascular and interstitial compartments under gain and loss situations. This equation formulates the physiological principle associated with steady-state change in BV response. In the steady-state, the intravascular compartment gains 1/(1 + *α*_*u*_) or loses 1/(1 + *α*_*v*_) fraction of the total infusion or loss, respectively. In other words, in the steady-state, the remaining *α*_*u*_/(1 + *α*_*u*_) or *α*_*v*_/(1 + *α*_*v*_) fraction transfers to or is removed from the interstitial compartment.

The feedback controller replicating inter-compartmental shift attempts to minimize the difference between change in BV response (Δ*BV*) and *r*_*BV*_, denoted by *e*_*BV*_ (*e*_*BV*_ = *r*_*BV*_ − Δ*BV*; see Fig 1A). The shift is computed by a proportional-integral (PI) response to *e*_*BV*_ at each time, driving the BV response to its desired state (see Fig 1B):

$$\begin{array}{c} q\left(t\right)=-K_{p}e_{BV}\left(t\right)-K_{i}\int_{0}^{t}e_{BV}\left(\tau \right)d\tau \end{array}$$ (2)

where *K*_*p*_ and *K*_*i*_ denote the controller’s proportional and integral gains. The direction of shift is determined by the sign of error, e.g., a negative *e*_*BV*_ sets a shift of fluid toward interstitial compartment (see Fig 1A).

By seeking the conservation of volume for the intravascular compartment, the rate of change in BV response can be obtained as follows:

$$\begin{array}{c} \Delta \dot{BV}\left(t\right)=U\left(t\right)-V\left(t\right)-q\left(t\right) \end{array}$$ (3)

Combining Eqs 1 to 3 leads us to the following equation for governing the BV response subject to fluid gain and loss:

$$\begin{array}{c} \begin{array}{c} \Delta \dddot{BV}\left(t\right)+K_{p}\Delta \ddot{BV}\left(t\right)+K_{i}\Delta \dot{BV}\left(t\right)=\ddot{U}\left(t\right)-\ddot{V}\left(t\right)+\frac{K_{p}}{1+\alpha_{u}}\dot{U}\left(t\right)\\ -\frac{K_{p}}{1+\alpha_{v}}\dot{V}\left(t\right)+\frac{K_{i}}{1+\alpha_{u}}U\left(t\right)-\frac{K_{i}}{1+\alpha_{v}}V\left(t\right) \end{array} \end{array}$$ (4)

#### Model 2: The refined BV model

The fluid distribution ratios *α*_*u*_ and *α*_*v*_ in the original model are assumed to be constant over the entire range of BV no matter how much the intravascular volume has expanded or compressed during fluid perturbation. Based on the physiological principles, however, the distribution ratio parameters can vary over time depending on the state of intravascular volume expansion or compression [22]. For example, looking toward the steady-state response to a same amount of fluid infusion, intravascular compartment can gain a larger fraction of fluid (smaller *α*_*u*_) when the BV is at a lower range. However, when the BV is at a higher range, the shift of fluid toward interstitial volume becomes more pronounced (larger *α*_*u*_) which avoids intravascular fluid overload. This motivate us to enhance the original model with a refined *r*_*BV*_ that is dynamically corrected over time as the state of BV response updates, even when no infusion or hemorrhage input is present (see the right picture in Fig 1C):

$$\begin{array}{c} \begin{array}{c} r_{BV}\left(t\right)=r_{BV,u}\left(t\right)+r_{BV,v}\left(t\right) \end{array} \end{array}$$ (5)

$$\begin{array}{c} \begin{array}{c} {\dot{r}}_{BV,u}\left(t\right)=A_{u}r_{BV,u}\left(t\right)+\frac{1}{1+\alpha_{u}}U\left(t\right),r_{BV,u}\left(0\right)=0 \end{array} \end{array}$$ (6)

$$\begin{array}{c} \begin{array}{c} {\dot{r}}_{BV,v}\left(t\right)=A_{v}r_{BV,v}\left(t\right)-\frac{1}{1+\alpha_{v}}V\left(t\right),r_{BV,v}\left(0\right)=0 \end{array} \end{array}$$ (7)

where *r*_*BV*,*u*_ and *A*_*u*_ denote the desired steady-state change in BV and state parameter due to infusion, while *r*_*BV*,*v*_ and *A*_*v*_ indicate the same parameters for the fluid loss. Using the revised model of *r*_*BV*_, the refined model is obtained as below:

$$\begin{array}{c} \begin{array}{c} \Delta \dddot{BV}\left(t\right)+K_{p}\Delta \ddot{BV}\left(t\right)+K_{i}\Delta \dot{BV}\left(t\right)=\ddot{U}\left(t\right)-\ddot{V}\left(t\right)+\frac{K_{p}}{1+\alpha_{u}}\dot{U}\left(t\right)\\ -\frac{K_{p}}{1+\alpha_{v}}\dot{V}\left(t\right)+\frac{K_{i}+K_{p}A_{u}}{1+\alpha_{u}}U\left(t\right)-\frac{K_{i}+K_{p}A_{v}}{1+\alpha_{v}}V\left(t\right)\\ +A_{u}\left(K_{p}A_{u}+K_{i}\right)r_{BV,u}\left(t\right)+A_{v}\left(K_{p}A_{v}+K_{i}\right)r_{BV,v}\left(t\right) \end{array} \end{array}$$ (8)

where *r*_*BV*,*u*_(*t*) and *r*_*BV*,*v*_(*t*) are shown in Eqs 6 and 7.

### Experimental data

Details about experimental data can be found in our prior study [23]. In short, the BV data were collected from 11 conscious sheep undergoing hemorrhage and fluid infusion. All the animals received Lactated Ringer’s solution (LR), a commonly used crystalloid fluid. In a separate study, 5 of the animals received Hextend (Hex), made from natural sources of starch. These 5 animals received LR and Hex in a randomized order, each 5 days apart from the last experiment.

Each study lasted for 180 min. After a baseline measurement, each study was started with a 25 ml/kg hemorrhage, lasted for 15 min. 15 min after the end of hemorrhage, fluid infusion was started and continued for 150 min, until the experiment ended. Rate of fluid administration was determined by a closed-loop controller described in a prior work [23]. Two additional 5 ml/kg hemorrhage was also applied 35 and 55 min after the end of first hemorrhage, each lasted for 5 min.

Baseline BV, *BV*(0),was measured in each animal using indocyanine green dye (ICG) [24]. Hematocrit (Hct), the ratio between the red blood cell (RBC) volume and BV, was measured every 5 or 10 min throughout the experiment and used to compute the change in BV at each measurement point *t*_*k*_:

$$\begin{array}{c} \begin{array}{c} \Delta BV\left(t_{k}\right)=BV\left(t_{k}\right)-BV\left(0\right)=RBC\left(t_{k}\right)+PV\left(t_{k}\right)-BV\left(0\right) \end{array} \end{array}$$ (9)

$$\begin{array}{c} \begin{array}{c} RBC\left(t_{k}\right)\approx RBC\left(0\right)+\Sigma_{i=1}^{k}\Delta RBC\left(t_{i}\right)=BV\left(0\right)H\left(0\right)-\Sigma_{i=1}^{k}V_{H}\left(t_{i}\right)H\left(t_{i}\right) \end{array} \end{array}$$ (10)

$$\begin{array}{c} \begin{array}{c} PV\left(t_{k}\right)\approx \left(1-H\left(t_{k}\right)\right)RBC\left(t_{k}\right)/H\left(t_{k}\right) \end{array} \end{array}$$ (11)

where *PV*, *H*, and *V*_*H*_ are plasma volume, Hct, and amount of blood withdrawn for hemorrhage, respectively.

### Model parameter estimation

The approach we take to learn the model parameters is the maximum-likelihood estimation (MLE), which finds the parameter values maximizing the likelihood of measured BV response to hemorrhage and fluid infusion. The original mathematical model includes 4 parameters to tune, i.e., {*α*_*u*_, *α*_*v*_, *K*_*p*_, *K*_*i*_}, while the refined model has two additional parameters, {*A*_*u*_, *A*_*v*_}. We first assess the identifiability of each model. Next, we define an optimization problem to determine the model parameters with the best fitting performance.

#### Structural identifiability analysis

*Original model*. In this section, we convert the original model to the frequency domain for simple assessment of the model identifiability. By taking the Laplace transform of Eq 4, the following equation is obtained:

$$\begin{array}{c} \Delta BV\left(s\right)={\left(\begin{array}{c} -K_{p}\\ -K_{i}\\ 1\\ K_{p}/\left(1+\alpha_{u}\right)\\ -K_{p}/\left(1+\alpha_{v}\right)\\ K_{i}/\left(1+\alpha_{u}\right)\\ -K_{i}/\left(1+\alpha_{v}\right) \end{array}\right)}^{T}(\begin{array}{c} s^{-1}\Delta BV\left(s\right)\\ s^{-2}\Delta BV\left(s\right)\\ s^{-1}\left(U\left(s\right)-V\left(s\right)\right)\\ s^{-2}U\left(s\right)\\ s^{-2}V\left(s\right)\\ s^{-3}U\left(s\right)\\ s^{-3}V\left(s\right) \end{array})=\Theta^{{}^{\prime }}\Phi \left(s\right) \end{array}$$ (12)

where *s* is the complex frequency variable with a unit of *sec*^−1^ [25]. Based on estimation theory [26], with a perturbation input that is rich in the frequency content and as a result by virtue of an informative data set Φ [27], the vector Θ becomes fully identifiable with a unique set of parameters. Simply put, *K*_*p*_ can be determined from the 1^st^ element, *K*_*i*_ from the 2^nd^, *α*_*u*_ from the 4^th^ and *α*_*v*_ from the 5^th^ element of Θ. Although the model identifiability is obvious from Eq 12, the model parameters should be obtained through an optimization problem for the best calibration results.

*Refined model*. We take the Laplace transform of the refined model. First, using Eqs 5–7, the following frequency domain equation is obtained for *r*_*BV*,*u*_ and *r*_*BV*,*v*_:

$$\begin{array}{c} r_{BV,u}\left(s\right)=\frac{1/(1+\alpha_{u})}{s-A_{u}}U\left(s\right) \end{array}$$ (13)

$$\begin{array}{c} r_{BV,v}\left(s\right)=-\frac{1/(1+\alpha_{v})}{s-A_{v}}V\left(s\right) \end{array}$$ (14)

By taking the Laplace transform of Eq 8 and using Eqs 13 and 14, the following equation is obtained in the frequency domain.

$$\begin{array}{c} \Delta BV\left(s\right)={\left(\begin{array}{c} A_{u}+A_{v}-K_{p}\\ K_{p}\left(A_{u}+A_{v}\right)-A_{u}A_{v}-K_{i}\\ -K_{p}A_{u}A_{v}+K_{i}\left(A_{u}+A_{v}\right)\\ -K_{i}A_{u}A_{v}\\ 1\\ -\left(A_{u}+A_{v}\right)+K_{p}/\left(1+\alpha_{u}\right)\\ \left(A_{u}+A_{v}\right)-K_{p}/\left(1+\alpha_{v}\right)\\ A_{u}A_{v}+\left(K_{i}-K_{p}A_{v}\right)/\left(1+\alpha_{u}\right)\\ -A_{u}A_{v}+\left(K_{p}A_{u}-K_{i}\right)/\left(1+\alpha_{v}\right)\\ -K_{i}A_{v}/\left(1+\alpha_{u}\right)\\ K_{i}A_{u}/\left(1+\alpha_{v}\right) \end{array}\right)}^{T}(\begin{array}{c} s^{-1}\Delta BV\left(s\right)\\ s^{-2}\Delta BV\left(s\right)\\ s^{-3}\Delta BV\left(s\right)\\ s^{-4}\Delta BV\left(s\right)\\ s^{-1}\left(U\left(s\right)-V\left(s\right)\right)\\ s^{-2}U\left(s\right)\\ s^{-2}V\left(s\right)\\ s^{-3}U\left(s\right)\\ s^{-3}V\left(s\right)\\ s^{-4}U\left(s\right)\\ s^{-4}V\left(s\right) \end{array})=\Theta^{{}^{\prime }}\Phi \left(s\right) \end{array}$$ (15)

Similarly, when subject to a fluid perturbation input with rich frequency content and thus, an informative data set Φ, the vector Θ is fully identifiable [27]. For instance, *K*_*p*_, *K*_*i*_, *A*_*u*_, and *A*_*v*_ can be determined by simultaneously solving 1^st^ to 4^th^ elements, *α*_*u*_ from either 6^th^, 8^th^, or 10^th^, and *α*_*v*_ from either 7^th^, 9^th^, or 11^th^ element. Again, for a better calibration performance, an optimization problem involving all the elements needs to be solved.

#### Practical identifiability analysis

A variance-based global sensitivity analysis [28] was performed to identify the behavior of model parameters subject to the range of estimated parameters and individual profiles of hemorrhage and fluid infusion. Unlike local sensitivity analysis methods, this method varies all parameters simultaneously and each variable is adjusted through its entire range identified across all individual subjects receiving LR or Hex. Once the range of each model parameter is identified (see the next subsection Parameter Estimation), each parameter is simulated 1000 times via a uniform distribution. The first-order or main effect *G* of *i*^*th*^ parameter on the BV response under individual input profile is computed through a Monte Carlo simulation:

$$\begin{array}{c} G_{i}=V_{i}/Var\left(BV\right) \end{array}$$ (16)

where *V*_*i*_ is the effect of *i*^*th*^ parameter on BV variation, leveraged over variation in other model parameters (refer to [29] for more details about calculating *V*_*i*_). It is standardized by the total variance in BV response to provide a fractional contribution. Therefore, the first-order effects across all the parameters should add up to 1. For better assessment, the sensitivity indices were reported at 4 different time instants: 30 min, 80 min, 120 min, and 180 min. Similar to prior studies [30–33], at each time, first-order sensitivity indices less than 0.01 are regarded as insensitive parameters. We also consider those larger than 0.1 as highly sensitive and the ones between 0.01 and 0.1 as sensitive parameters. A parameter whose sensitivity index is less than 0.01 at all time instants is considered insignificant and might be removed with minimal degradation in the model performance.

#### Parameter estimation

The models presented in the last section provide an approximation of BV response over time

$$\begin{array}{c} \Delta BV\left(t_{k}\right)=\hat{\Delta BV}\left(t_{k}\right)|\Theta +\epsilon \left(t_{k}\right) \end{array}$$ (17)

where *k* = 1, …*K* is the time index for *K* successive discrete observations, $\hat{\Delta BV}\left(t_{k}\right)|\Theta$ is the model reproduced BV response for a chosen Θ, and *ϵ*(*t*_*k*_) is the error between the model output and BV observations over time. Assuming that *ϵ*(*t*_*k*_) is distributed normally, independently, and identically with mean *E*(*ϵ*(*t*_*k*_)) = 0 and variance *V*(*ϵ*(*t*_*k*_)) = *σ*^2^ for all *t*_*k*_, the following equation is defined according to a normal distribution.

$$\begin{array}{c} N\left(\epsilon ;0,\sigma^{2}\right)=\frac{1}{\sqrt{2\pi \sigma^{2}}}\exp\left\{\frac{-1}{2\sigma^{2}}{\left(\Delta BV\left(t_{k}\right)-\hat{\Delta BV}\left(t_{k}\right)|\Theta \right)}^{2}\right\} \end{array}$$ (18)

Therefore, the observations Δ*BV*(*t*) have a density function $N(\Delta BV;\hat{\Delta BV(t)}|\Theta ,\sigma^{2})$ which are the same form as those of the *ϵ*. Thus, the likelihood function of Θ and *σ*^2^, based on the sample, is

$$\begin{array}{c} \begin{array}{c} L\left(\Theta ,\sigma \right)=\prod_{k=1}^{K}N\left(\Delta BV\left(t_{k}\right);\hat{\Delta BV}\left(t_{k}\right)|\Theta ,\sigma^{2}\right)\\ ={\left(2\pi \sigma^{2}\right)}^{-K/2}\exp\left\{\frac{-1}{2\sigma^{2}}{\left(\Delta BV\left(t_{k}\right)-\hat{\Delta BV}\left(t_{k}\right)|\Theta \right)}^{{}^{\prime }}\left(\Delta BV\left(t_{k}\right)-\hat{\Delta BV}\left(t_{k}\right)|\Theta \right)\right\} \end{array} \end{array}$$ (19)

The log-likelihood function L* is obtained by taking the logarithm of Eq 19.

$$\begin{array}{c} \begin{array}{c} L^{*}\left(\Theta ,\sigma \right)=-\frac{K}{2}\ln\left(2\pi \right)-\frac{K}{2}\ln\left(\sigma^{2}\right)\\ -\frac{1}{2\sigma^{2}}{\left(\Delta BV\left(t_{k}\right)-\hat{\Delta BV}\left(t_{k}\right)|\Theta \right)}^{{}^{\prime }}\left(\Delta BV\left(t_{k}\right)-\hat{\Delta BV}\left(t_{k}\right)|\Theta \right) \end{array} \end{array}$$ (20)

To identify the model parameters, an optimization problem is defined

$$\begin{array}{c} \mu^{ML}=\left\{\Theta^{*},\sigma^{*}\right\}=\text{arg}\;\underset{\mu }{\text{min}}(-L^{*}\left(\Theta ,\sigma \right)+{2\gamma ∥\Theta ∥}_{2}) \end{array}$$ (21)

where *γ* > 0 is the *L*_2_ penalty term, ‖.‖_2_ denotes the *L*_2_ norm operator and penalizes the L2 norm of the model parameters to force them to be small with reduced estimation variance, thus to avoid over-fitting. Using Δ*BV* measured for a given subject, solution to the optimization problem 21 minimizes the negative log-likelihood and derive the set of optimal parameters, i.e., {*α*_*u*_, *α*_*v*_, *K*_*p*_, *K*_*i*_} for the original and {*α*_*u*_, *α*_*v*_, *K*_*p*_, *K*_*i*_, *A*_*u*_, *A*_*v*_} for the refined model, leading to a fully individualized model with minimum discrepancy between the true versus model-reproduced BV response.

We tuned the regularization parameter *γ* > 0 for each data-set using an inner-level validation procedure. We used three different penalty levels, *γ* > 0 ∈ {1, 3, 5}, identified the parameters for both models using the first 120 min of the data, and reproduced the remaining 60 min. For each model, the penalty with the least AIC value (see Eq 23) was selected and used in solving the optimization problem (Eq 21).

### Model evaluation

We compared the original and refined models in terms of their calibration performance and predictive capability. For a comprehensive assessment, the models were examined through different tools and techniques explained below.

#### Model calibration assessment

We used data from 16 animals (11 LR and 5 Hex) to identify 16 individualized models. For each subject, we utilized measured BV data and solved Eq 21 to identify the optimum set of parameters for each subject. The model reproduced BV response was then compared with its true counterpart and the following measures were reported:

*Root-mean-squared error*. RMSE is the square root of the second moment of the estimation error, which aggregates the estimation error for various times into a single value, computed by

$$\begin{array}{c} \begin{array}{c} RMSE=\sqrt{\frac{\Sigma_{i=1}^{K}{\left(\Delta BV\left(t_{i}\right)-\hat{\Delta BV}\left(t_{i}\right)\right)}^{2}}{K}} \end{array} \end{array}$$ (22)

*Akaike information criterion*. AIC is a model selection tool and deals with the trade-off between model’s accuracy and complexity to prevent over-fitting. AIC seeks a model that has a good fit to the true data but with fewer parameters, and is defined as

$$\begin{array}{c} \begin{array}{c} AIC=-2L^{*}+2P \end{array} \end{array}$$ (23)

where L* is the log-likelihood function defined in Eq 20, and P is the number of model parameters, 4 in the original and 6 in the refined model.

*Multi-dimensional measure*. Mean-squared error is the sum of variance and square of the bias observed in an estimation. As a result, RMSE outputs a trade-off between variance and bias but not their segregated values [34]. A comprehensive fitting assessment, however, requires to examine the adequacy of multiple estimation features individually defined over time and the range of the data. For instance, an underfitting occurs when the model shows low variance but high bias, while overfitting leads to a fit with high variance and low bias [35]. Therefore, a joint measure of features cannot completely determine the extent to which a model captures the underlying structure of the data. Here, we developed a tool that considers multiple individual features of a model calibrated to each subject to examine the fitting performance between the two models. We defined four features, bias, standard error of residuals, trend of error over time, and trend of error over the BV range. We fitted both models to each individual subject using Eq 21 and collected the features for both original and refined models. We then normalized the features to have values between 0 and 1 across all the subjects. Next, we statistically compared the features in a 4-dimensional space in Cartesian coordinates. In addition, for each individual fit, we identified the Euclidean distance as a distance between the origin and the points corresponding to the normalized features

$$\begin{array}{c} U_{d}=\sqrt{\sum_{i=1}^{4}f_{i}^{2}} \end{array}$$ (24)

where *U*_*d*_ and *f* denote the Euclidean distance and features, respectively. We identified and compared the euclidean distance values across the original and refined models.

#### Predictive capability assessment

**Fig 2:**
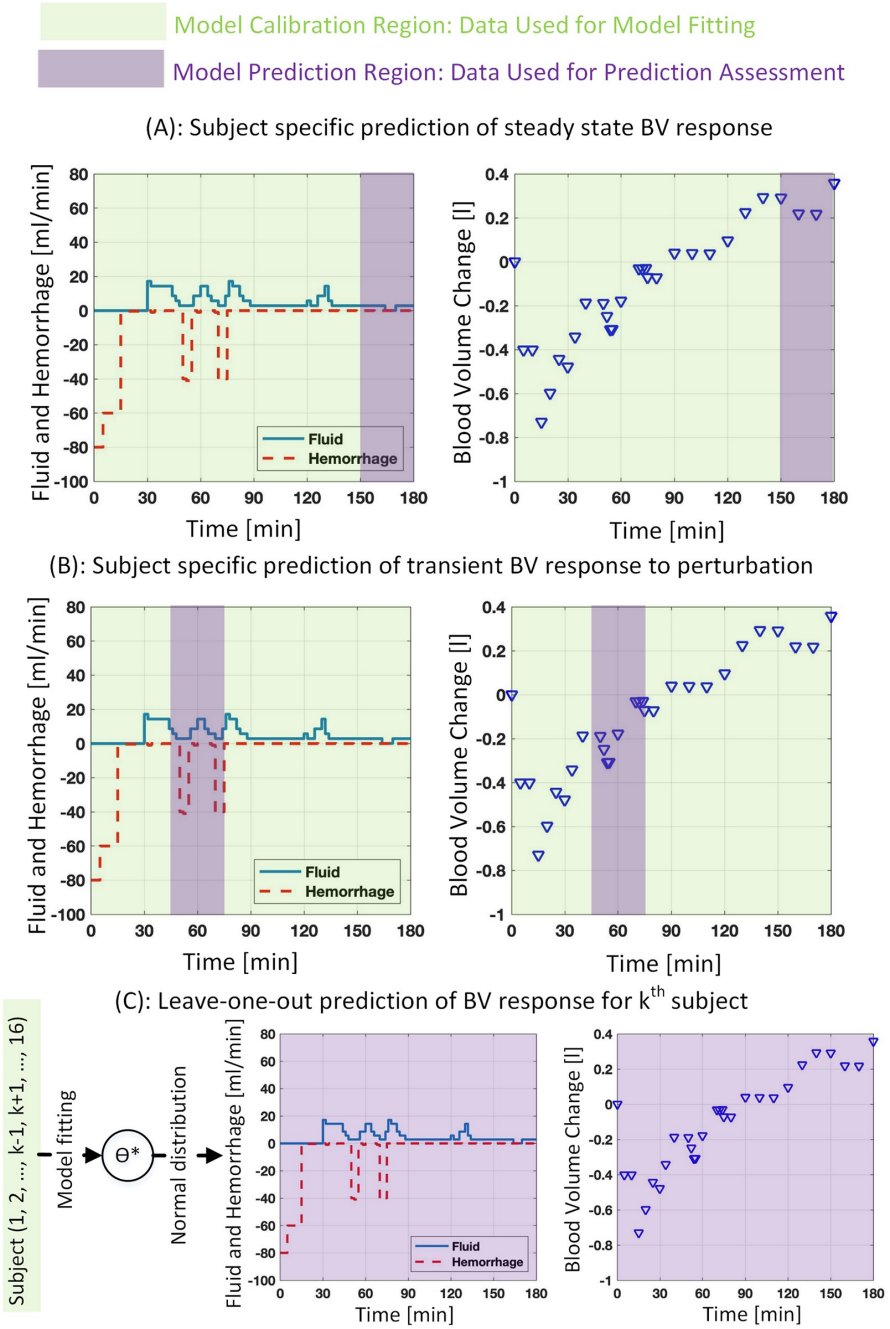
Predictive capability assessment under three scenarios, (A): subject specific prediction of steady-state BV response (150^+^-180 min) via bootstrapping the rest of data (0-150 min), (B): subject specific prediction of transient BV response to second and third hemorrhage perturbations (45-80 min) via bootstrapping the rest of data (0-45^−^ and 80^+^-180 min), (C): Leave-one-out prediction of BV response in each subject using the normal distribution of parameters identified in other subjects (for more details please refer to text). The distribution of *α*_*u*_ in the original model and *A*_*u*_ in the refined model was identified based on LR or Hex, depending on whether the subject received LR or Hex, and not a combination of both LR and Hex due to their distinct dispersion. Green and purple colors show the data used for model calibration and prediction assessment, respectively.

Besides the calibration performance assessment described above, it is important to quantitatively examine the extent to which a mathematical model is an adequate representation of physiological measurements under conditions for which the model has not been calibrated, as the intended use inputs and boundary conditions can be different from those in the calibration data. In addition, a model fitted to a physiological variable may be judged as fit for purpose and may not adequately work against new data, even those obtained from the same subject, in the same environment, and for a similar intended application [36]. Therefore, accuracy assessment of a predicted response by an interpolation or extrapolation of the model to diverse inputs and boundary conditions is essential. To include a wide range of fluid perturbation conditions for predictive capability performance assessment, we examined the model response prediction under three different scenarios, shown in Fig 2. First, we assessed the adequacy of subject-specific predictions for the steady-state BV responses, during the last 30 min of each experiment, and through an extrapolation of the model response that was calibrated to a sub-sample of data occurred earlier (upper panel in Fig 2). We considered this as steady-state response prediction since no hemorrhage took place during this time and the amount of inputted fluid and BV variation were much less compared to those in earlier times. Second, we assessed the subject-specific predictions for a transient BV responses during 45-80 min, which includes the second and third hemorrhage events, through an interpolation of the model response calibrated to a sub-sample of the remaining data, i.e., those outside of 45-80 min time range (middle panel in Fig 2). Finally, we assessed the entire BV response prediction for each individual through a leave-one-out procedure, where the model parameters were selected based on their values identified from all other subjects (lower panel in Fig 2). These three scenarios are very distinct and replicate different input profiles—e.g., low and large fluid perturbation in the steady-state and transient prediction scenarios—and boundary conditions, where the leave-one-out procedure intends a new subject with different treatments and environmental uncertainty as compared to others. It is noted that the effects of sensor inaccuracy have not been explicitly considered in this study. BV response measurements are used for predictive performance evaluation, assuming that the effects of noise in measurements are included in the reported prediction interval in the Results section.

*(A) Subject-specific prediction of steady-state BV response*. The 95% confidence interval for model-predicted steady-state BV response was obtained through a sub-sampling procedure, known as bootstrapping. For each individual, we selected a 75% sub-sample of BV data collected in 0-150 min time range and fitted both models to the sub-sampled data. We used a 75% sub-sample to avoid the large parameter estimation uncertainty and keep the number of experimental data points at least 4 times as large as the number of model parameters. Next, the identified models were used to predict the steady-state BV responses occurred after 150 min till the end of experiment. We repeated this procedure for 100 times (it is noted that the bootstrapping results remained unchanged by doing this for more than 100 times). After collecting all predicted responses, we used the percentile method for constructing the 95% confidence interval, which included all the predictions between the 2.5^*th*^ and 97.5^*th*^ percentiles of the forecast at each prediction time.

After collecting the 95% confidence interval, we measured and compared predictive capability of the two mathematical models by evaluating their forecasts. In this work, we used the interval score developed in [37], that measures a confidence interval using a performance score. The score rewards a forecast for a narrower prediction envelope, while applies a penalty, the magnitude of which depends on a significance level *α*, when the measurement misses the confidence interval. For a prediction point *t*_*k*_, if $\Delta \hat{BV}{\left(t_{k}\right)}^{l}$ and $\Delta \hat{BV}{\left(t_{k}\right)}^{u}$ are the lower and upper (1 − *α*) × 100% prediction envelope and Δ*BV*(*t*_*k*_) is the true measurement, the interval score is defined as

$$\begin{array}{c} S\left(t_{k}\right)=\{\begin{array}{cc} \Delta \hat{BV}{\left(t_{k}\right)}^{u}-\Delta \hat{BV}{\left(t_{k}\right)}^{l}+\frac{2}{\alpha }\left(\Delta \hat{BV}{\left(t_{k}\right)}^{l}-\Delta BV\left(t_{k}\right)\right), & \Delta BV\left(t_{k}\right)<\Delta \hat{BV}{\left(t_{k}\right)}^{l}\\ \Delta \hat{BV}{\left(t_{k}\right)}^{u}-\Delta \hat{BV}{\left(t_{k}\right)}^{l}+\frac{2}{\alpha }\left(\Delta BV\left(t_{k}\right)-\Delta \hat{BV}{\left(t_{k}\right)}^{u}\right), & \Delta \hat{BV}{\left(t_{k}\right)}^{u}<\Delta BV\left(t_{k}\right)\\ \Delta \hat{BV}{\left(t_{k}\right)}^{u}-\Delta \hat{BV}{\left(t_{k}\right)}^{l}, & \text{Otherwise} \end{array} \end{array}$$ (25)

In this study, the significance level *α* is considered to be 0.05. The identified scores were compared between the two models using statistical methods. We also compared the proportion of measurements that were within the prediction envelope, denoted as *PM*, for both original and refined models.

*(B) Subject-specific prediction of transient BV response to perturbation*. The 95% confidence interval for model predicted BV response to the second and third hemorrhage events was obtained through a sub-sampling procedure. For each individual, the BV response was predicted in the 45-80 min time span, using a model fitted through 75% sub-sample of the remaining BV data. This procedure was repeated for 100 times. The percentile method was used to construct the 95% confidence interval at each prediction point. The forecast interval performance at each prediction point was measured using the interval score defined in Eq 25. The identified scores as well as proportion of measurements within the prediction envelope were compared between the two models using statistical methods.

*(C) Leave-one-out prediction of BV response*. We predicted the BV response for each individual using both original and refined mathematical models and tuned parameters in other subjects. In each subject, a sample of size 1000 was generated for each parameter through a normal distribution identified from the tuned parameters in other subjects and within the lower and upper bound of these parameters. Except the *α*_*u*_ in the original model and *A*_*u*_ in the refined model, normal distribution of a parameter was identified from its values across other 15 subjects, while the lower and upper bound were minimum and maximum values across the range of the parameter in those 15 subjects. Normal distribution of *α*_*u*_ in the original or *A*_*u*_ in the refined model was determined solely based on the parameter values in LR or Hex subjects, depending on type of fluid given to the subject, and not their combination due to the distinct dispersion of the parameter between two groups (for more details please see Table 1 in the Results section). Since no clear correlation was seen between each pair of parameters (original model *r*^2^ = 0.11±0.14, refined model *r*^2^ = 0.15±0.15), all the parameters were allowed to freely change within their range, i.e., regardless of variation in other parameters, and based on their identified distribution. The leave-one-out BV response confidence intervals were predicted through 1000 simulations, the parameters of which were randomly selected from the normally distributed samples. The percentile method was used to construct the 95% confidence interval at each point. The performance of the model forecast at each time was measured through interval score and proportion of measurements within the prediction envelope and examined between the two models.

**Table 1:** Calibrated parameters for the original and refined models across LR and HEX data, along with the *P*-values (2-sample unequal t-test) reported between the identified parameters for each model. Underline bold numbers indicates significant difference between the parameters for LR and HEX data. The calibrated parameters for each subject are provided in the Supporting information section.

	*A*_*u*_	*A*_*v*_	*α*_*u*_	*α*_*v*_	*K*_*p*_	*K*_*i*_
Original Model (LR)	-	-	1.72±0.66	1.06±0.78	0.08±0.04	0.003±0.001
Original Model (HEX)	-	-	-0.18±0.31	0.91±0.46	0.13±0.08	0.007±0.004
P-value (Original)	-	-	**2e-6**	0.63	0.25	0.09
Refined Model (LR)	-0.16±0.23	-0.007±0.006	0.20±0.69	0.60±0.47	0.28±0.36	0.01±0.01
Refined Model (HEX)	-0.004±0.006	-0.006±0.005	-0.06±0.27	0.64±0.30	0.19 ±0.14	0.01±0.003
P-value (Refined)	**0.04**	0.63	0.30	0.83	0.47	0.57

## Results

In this section, we first present the identified parameters in both mathematical models and show how the identified parameters differ between LR and Hex. Next, the calibration performance is presented and compared between the two models. Finally, predictive capability assessments are shown and examined in both models.

### Parameter estimation and identifiability analysis

Original and refined model parameters were identified by solving Eq 21 in each individual subject. Table 1 shows the averaged identified parameters grouped per model and fluid type. Statistical test performed for each parameter and across fluid types shows that the mean of only one parameter in each model is significantly different between LR and Hex: *α*_*u*_ in the original model (*P* = 2 × 10^−6^, 2-sample unequal t-test, LR: *N* = 11 and HEX: *N* = 5); and *A*_*u*_ in the refined model (*P* = 0.04, 2-sample unequal t-test, LR: *N* = 11 and Hex: *N* = 5). Given that the models have different dynamic characteristics and mechanisms in updating the reference blood volume, a comparison between the common parameters of the two models was not made.

Using the identified range of parameters, global sensitivity indices were computed using Eq 16 and a Monte Carlo simulation. The sensitivity indices were averaged across all 16 subjects and reported at 30 min, 80 min, 120 min, and 180 min.

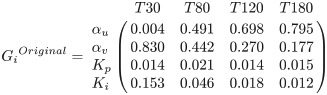
(26)

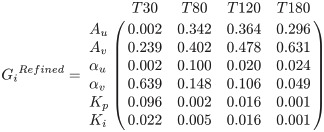
(27)

It is clear that all parameters in both models remain significant as there is no parameter with a sensitivity index less than 0.01 at all time instants. At time 30 min, before the fluid infusion is initiated, *α*_*u*_ in the original model and *A*_*u*_ and *α*_*u*_ in the refined model are insensitive, while *α*_*v*_ in the original model and *A*_*v*_ and *α*_*v*_ in the refined model remain highly sensitive. At time instants greater than 30 min and when fluid has started to be given to subjects, *α*_*u*_ in the original model and *A*_*u*_ in the refined model become highly sensitive, and *α*_*u*_ in the refined model turn to be sensitive, all supporting the adequacy of the results. In addition, *K*_*p*_ and *K*_*i*_, those in the refined model in particular, are mostly sensitive at 30 min, when the rate of fluid shift between intravascular and interstitial compartments is presumably maximal.

#### Model calibration assessment

**Fig 3:**
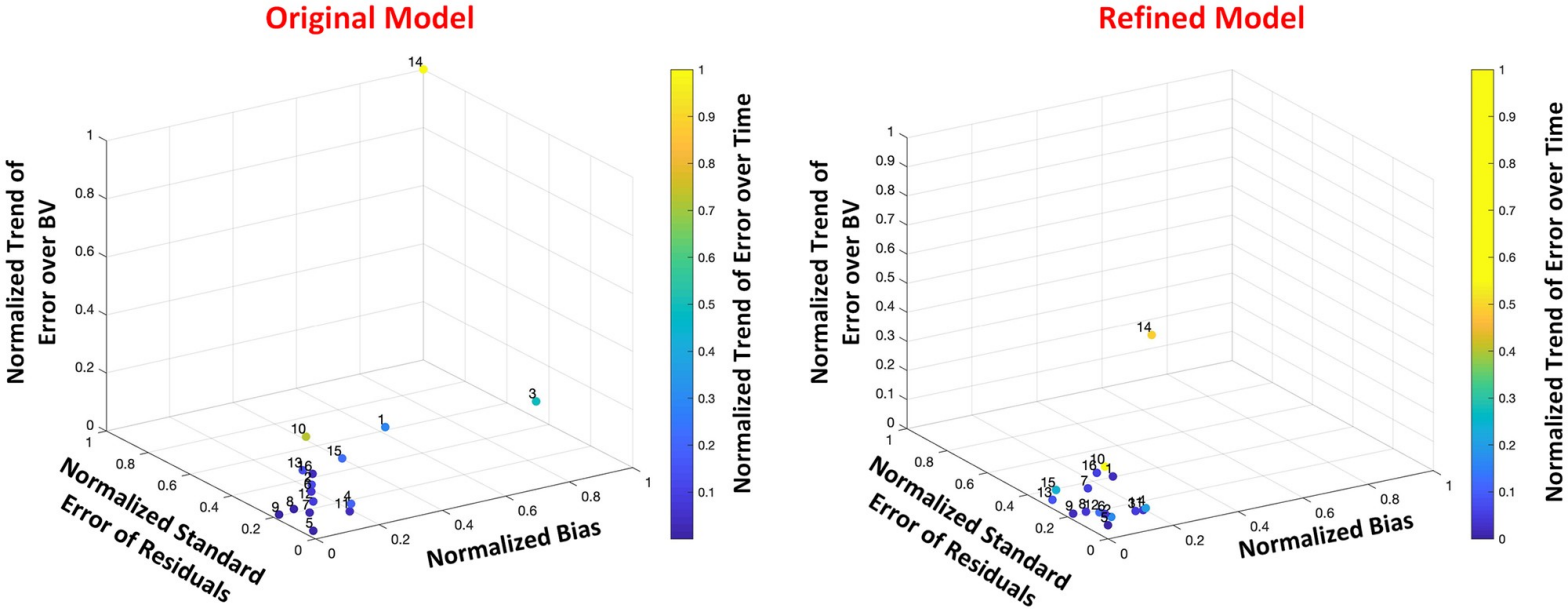
Multi-dimensional measure shown for 16 individuals in both original (left) and refined (right) mathematical models. As compared to the original model, the points in the refined model are significantly closer to the origin in terms of bias, standard error of residuals, and trend of error over the BV range (*P* < 0.04, paired t-test, *N* = 16), while comparable trend of error over time is observed between the two models (*P* = 0.08, paired t-test, *N* = 16). Averaged Euclidean distance from each point is also significantly smaller in the refined model (see Table 2, *P* = 0.02, paired t-test, *N* = 16).

Table 2 compares the goodness-of-fit and calibration performance between the two models using three different methods: RMSE, AIC, and multi-dimensional measure. Averaged among all individuals, the results show that the RMSE is significantly lower in the refined model (see Table 2, *P* = 0.03, paired t-test, *N* = 16). In addition, the refined model shows comparable AIC to that of the original model, as among all 16 subjects, the frequency in which each model attained the minimum AIC is equal, i.e., each model is suggested as the better model in 8 subjects (see Table 2), indicating the better calibration performance is not due to over-fitting the BV data. Fig 3 shows the multi-dimensional measure in a 4-dimensional space, where the Euclidean distance for each point, i.e., each individual, can be obtained as the distance between the point from the origin. As it can be seen from the figure, the points in the refined model are scattered in closer vicinity of the origin as compared to the original model. This is also evidenced from Table 2, where among the multi-dimensional error features in the 4-D space, bias, standard error of residuals, and trend of error over the BV range are significantly smaller in the refined model (*P* < 0.04, paired t-test, *N* = 16), while a comparable trend of error over time is seen between the two models (*P* = 0.08, paired t-test, *N* = 16). In addition, the Euclidean distance is significantly smaller in the refined model (*P* = 0.02, paired t-test, *N* = 16). In summary, the results show that the calibration performance is significantly enhanced in the refined model.

**Table 2:** Model calibration assessment based on root-mean-squared error (RMSE), Akaike information criterion (AIC) and the frequency in which each model attained the minimum AIC (FR), and multi-dimensional measure. Regarding the multi-dimensional measure, 4-D features, i.e., bias, standard error of residuals, trend of error over time, and trend of error over the BV range, as well as the Euclidean distance defined in Eq 24 are computed and compared between the two models. Results show that the refined model has significantly better performance in terms of RMSE and multi-dimensional measure. In addition, the models are comparable in terms of AIC, where among all 16 subjects, the frequency in which each model attained the minimum AIC is equal, i.e., 8. P-values are obtained using paired t-test. Underline bold numbers indicates significant difference between calibration performance of the two models.

	RMSE	AIC (FR)	Multi-dimensional 4-D features & Euclidean distance
Original Model	60.4±73.2	-96.5±36.2 (8)	[0.24±0.29,0.24±0.23,0.14±0.24,0.22±0.29] & 0.46±0.49
Refined Model	55.4±68.1	-100±35.1 (8)	[0.11±0.16,0.20±0.19,0.06±0.07,0.14±0.17] & 0.31±0.27
P-value	**0.03**	0.06	**0.03±0.03**& **0.02**

#### Predictive capability assessment

**Fig 4:**
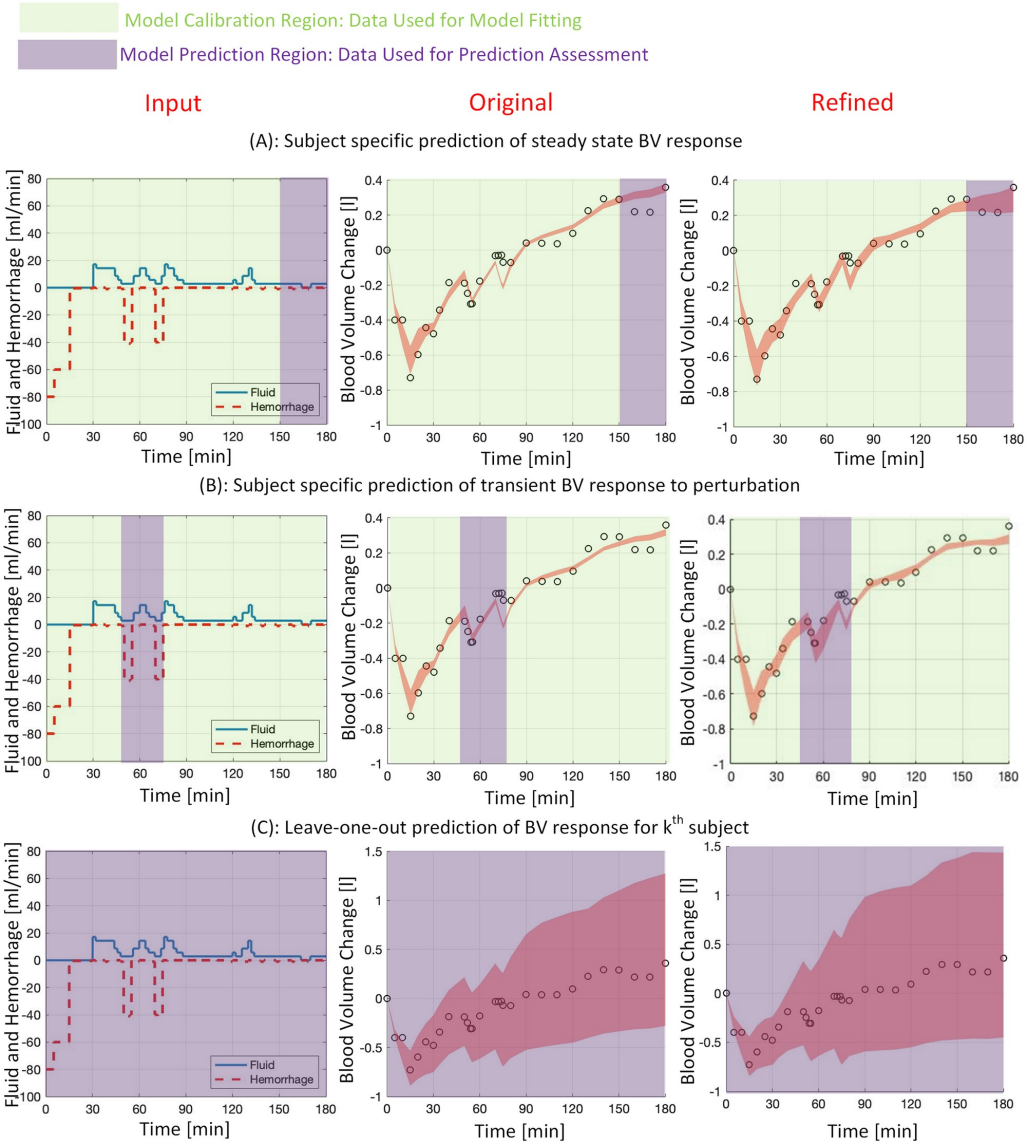
Predictive capability assessment across the original (middle column) and refined (right column) mathematical models under three scenarios. A) upper panel: predicting the steady-state response (150-180 min) by fitting a 75% sub-sample of BV measurement between 0-150 min, B) middle panel: predicting transient response (45-80 min) by fitting a 75% sub-sample of BV measurement between 0-45 and 80-180 min, C) lower panel: leave-one-out prediction of the inter-subject response using a normal distribution of identified parameters in other individuals.

Fig 4 shows the predictive capability assessment for both models and under 3 different scenarios for a representative subject. In the first scenario, shown in the upper panel, the steady-state response (150-180 min) was predicted by fitting a 75% sub-sample of the remaining data. As it can be seen, the prediction envelope of the refined mathematical model (right column) is much closer to the measurements and includes two out of three measurements (i.e., the last two data points in the prediction region), while the prediction envelope of the original model (middle column) includes only one measurement (i.e., the last data point in the prediction region). In the second scenario, shown in the middle panel, the transient response to the second and third hemorrhage events was predicted by fitting a 75% sub-sample of data in 0-45 min and 80-180 min range. It can be seen from Fig 4 that the prediction envelope in the refined model is closer to the measurement, where it includes four out of nine measurements (i.e., the first four data points in the prediction region), as compared to one measurement in the original model (i.e., the fourth data point in the prediction region). In the third scenario, shown in the lower panel, the prediction envelope is obtained through a leave-one-out procedure. It is seen from the figure that the prediction envelope corresponding to the refined model includes one more measurement compared to the original model. The refined model, however, produces a slightly wider prediction envelope after 60 min, leading to slightly larger interval scores. Averaged across all individuals, Table 3 compares the predictive capability performance between the two models and under the three scenarios. The results indicate that the original and refined models similarly perform in the steady-state response prediction, whereas the means of interval score and proportion of measurements within the prediction envelope do not show a significant difference (interval score: *P* = 0.3, paired t-test, *N* = 45; proportion of measurements: *P* = 0.8, Chi-squared test, *N* = 45). The refined model shows significantly better performance in transient response prediction, whereas both the interval score and proportion of measurements within the prediction envelope are significantly better in the refined model (interval score: *P* = 7 × 10^−4^, paired t-test, *N* = 127; proportion of measurements: *P* = 0.02, Chi-squared test, *N* = 127). Finally, the interval score for the leave-one-out procedure was comparable in both models (*P* = 0.8, paired t-test, *N* = 438). However, significantly larger number of measurements were included in the refined model’s prediction envelope (*P* = 3 × 10^−3^, Chi-squared test, *N* = 438).

**Table 3:** Predictive capability assessment for different prediction scenarios in both original and refined models. *S* and *PM* indicate the prediction interval score and proportion of measurements within the prediction envelope and are reported for prediction of steady-state BV response (150-180 min, i.e., *S*_150 − 180_ and *PM*_150−180_), prediction of transient response (45-80 min, i.e., *S*_45−80_ and *PM*_45−80_), and leave-one-out prediction (i.e., *S*_*Leave*-*One*-*Out*_ and *PM*_*Leave*-*One*-*Out*_). P-values for the *S* and *PM* are obtained using paired t-test and Chi-squared test, respectively. Underline bold numbers indicates significant difference between the two models.

	*S*_150−180_	*PM*_150−180_	*S*_45−80_	*PM*_45−80_	*S*_*Leave*−*One*−*Out*_	*PM*_*Leave*−*One*−*Out*_
Original Model	2.4±3.6	24%	2.1±2.4	28%	1.0±1.6	88%
Refined Model	2.7±3.8	27%	1.7±2.4	43%	1.0±1.3	94%
P-value	0.34	0.81	**7e-4**	**0.02**	0.79	**3e-3**

## Discussion

Toward the goal of establishing methods to compare models intended for use in PCLC medical device assessment and determine a mathematical model that better represents the physiological measurement, we proposed a pathway that can be potentially used as a guide for physiological model selection between candidate mathematical models. As a case study, we compared two physiological models for BV response to fluid perturbation: the original model that was developed in our prior work [19, 20], and a refined model that was built in this research, based on an expansion to the original model. This research focuses on first-principle physiological —as opposed to black-box— models aimed for PCLC medical device assessment. Our methods, therefore, should assess the models in terms of their physiological transparency and allow to compare the modeling performance in both calibration as well as predictive capability. We formulated a maximum likelihood estimation problem to separately identify the parameters for each model. We compared the calibration performance in terms of RMSE, AIC, and multi-dimensional measure. In addition, the predictive capability of the models were assessed under three different scenarios to investigate the adequacy of their predicted response under different input profiles and hemodynamic conditions. To compare the predictive capability, we used two measures: interval score (*S*) [37] and the proportion of measurements within the prediction envelope (*PM*). Based on the results, the refined mathematical model turned out to better represent the BV physiological data since it (1) significantly improved individualized model calibration performance to the experimental data, (2) did not increase the risk of overfitting despite its larger number of parameters compared to the original model, and (3) showed significantly enhanced predictive capability in subject-specific transient response prediction to hemorrhage, while at least comparable performance in steady-state and leave-one-out predictions. Taken together, the proposed mathematical approach can serve as a tool to select the best between multiple candidate physiological models for evaluation of PCLC medical devices.

### A physiological model should be identifiable and transparent

A model to be used for performance evaluation of a PCLC medical device should be identifiable, that is, the model parameters can be uniquely estimated under the input and boundary conditions relevant to the intended use of a device. Based on Eqs 12 and 15, it was shown that similar to the original model, the refined mathematical model which involves more parameters is theoretically identifiable. In addition, the optimization problem in Eq 21 was solved using different initial conditions for the parameters, where the identified parameters remained unchanged, further supporting the identifiablity of both models. Furthermore, based on Eqs 26 and 27, it was shown that all parameters in both models are significant, as their global sensitivity indices are not less than 0.01 at all the reported time instants.

Regarding the model transparency, both models are expected to be physiologically interpretable. In the original model, *α*_*u*_ denotes the steady-state distribution of fluid between intravascular and interstitial compartments under fluid administration. Unlike LR, Hex is made of high-molecular-weight hydroxyethyl starch 6% in isotonic saline, causing the body to keep the fluid longer in the intravascular space rather than leaving through its vascular endothelium [38]. Therefore, it is anticipated to have a smaller *α*_*u*_ for Hex compared to LR, as seen in Table 1 (*P* = 2 × 10^−6^). The original model dictates the reference BV (*r*_*BV*_) to update only during fluid administration, as shown in Eq 1. However, *r*_*BV*_ can change continuously over time, even in the absence of fluid administration. Indeed, it is an individual’s physiology that mostly determines the dynamics of change in BV response over time, so much as when no infusion is present. Motivated by this, the refined physiological model is built based on the concept that patient’s physiology can continuously alter the target state of BV, due to the dynamics of change in the colloid osmotic concentration in plasma [22]. Table 1 indicates that, while *α*_*u*_ in the refined model is still slightly smaller under Hex administration (*P* = 0.3), it is the state parameter *A*_*u*_ and it’s dynamical impact on *r*_*BV*_ —through subject’s physiology— that constructs the basis of difference in BV response to Hex and LR fluids (*P* = 0.04). Based on Eq 13, a smaller *A*_*u*_ leads to a larger low-frequency (DC) gain, amplifying the steady-state BV response under Hex administration. All together, *A*_*u*_ in the refined model represent a physiological mechanism modulating the target BV state under different fluid types.

### Selected model offers enhanced model calibration

The refined mathematical model can serve as a better model in terms of the model calibration performance. While it is important for a model to adequately capture the dynamics of change in physiological data used to infer the model parameters, the model should not be overly complex to avoid over-fitting. In this work, we assess the adequacy of data calibration using RMSE, AIC to evaluate the trade-off between complexity and accuracy of each model, and a new multi-dimensional measure that compares the adequacy of 4 major estimation features individually defined over time and range of the data. First, the results reported in Table 2 show that, compared to the original model, the refined model has significantly smaller RMSE (*P* = 0.03). It is noted that the RMSE in both original and refined models have a large standard deviation, which is due to large calibration error seen in one of the animal subjects —subject number 14 shown in Fig 3. Second, the models are comparable in terms of AIC, where the frequency in which each model attained the minimum AIC is equal. This indicates that enhanced calibration performance in the refined model is not trivial and not obtained due to over-fitting the BV data. Instead, the calibration performance improvement is based on the physiological model refinement and inclusion of the dynamic behavior of BV response to fluid perturbation. Finally, the multi-dimensional measure in the refined model is significantly smaller than the original model (*P* = 0.02), as seen in Fig 3. The figure clearly depicts how error features in the refined model are scattered in closer vicinity of the origin, leading to a significantly smaller average Euclidean distance from the origin to each point. It is noted that based on the figure and results shown in Table 2, the refined model significantly improves the calibration performance in terms of three estimation error features; bias, standard error of residuals, and trend of error over the BV range (*P* < 0.04), while the trend of error over time is comparable between the two models (*P* = 0.08). All together, the results indicate the suitability of the refined model in fitting BV data.

### Selected model offers enhanced predictive capability performance

The refined mathematical model performs better in terms of predictive capability against physiological states and under conditions to which the model has not been calibrated. This has been shown based on the predictive capability performance obtained under three different scenarios. The performance was measured via *S* score and proportion of measurements within the prediction envelope. As it is seen from Table 3, refined and original models showed comparable prediction performance in the steady-state BV response scenario, where *S* score as well as proportion of measurement that were within the prediction envelope were not significantly different (*P* > 0.34). Enhanced prediction performance of the refined model was the most evident in the transient BV response scenario, where the *S* score was significantly smaller (*P* = 7 × 10^−4^) and larger proportion of data points were within the generated prediction envelope (*P* = 0.02). While some of this discrepancy in prediction performance is due to different quality of calibration via training data (see results on calibration performance), a model with larger number of parameters can inherently explore a broader range of patient heterogeneity, named patient cohorts, thus, constructing a wider prediction envelope. As a result, the refined model has led to a broader prediction envelope to include a larger number of measurements, i.e., 15%, but not too wide to get a larger *S* score than the original model. Similarly, the refined model led to a significantly larger proportion of measurement within the prediction envelope for the leave-one-out scenario (*P* = 3 × 10^−3^). However, due to the same reason mentioned earlier, *S* score was identified to be similar between the two models (*P* = 0.79). Taken together, the refined model performed significantly better in the transient BV response and leave-one-out scenarios, while similar performance was seen for the steady-state response prediction. It is noted that the *S* scores identified in this study are obtained for a 0.05 significance level. A smaller significance level will benefit the refined model as its prediction envelopes contain a larger percentage of measurements. These evaluation results obtained by the use of experimental data suggest that for future evaluation of PCLC medical devices for fluid resuscitation, the refined model may lead to more realistic virtual patient cohorts for inputs and boundary conditions to which the model is not calibrated. Therefore, it may provide more credible results when testing PCLC medical device performance.

### Study limitation

This research provide a comparison pathway to select a model between a set of candidate models. As a case study, we examined the proposed pathway for two physiological models of BV response to fluid perturbation, intended for fluid resuscitation PCLC medical devices. This study, however, needs to be extended to other physiological domains, e.g., respiratory treatment, that are outside of the scope of the models in this work, or when patients go under multiple interactive PCLC medical devices. In addition, we studied the models that correspond only to the physiological response, while the effects of sensor inaccuracy or input disturbance has not been explicitly considered. Instead, data measured from the animal subjects that may have been contaminated by noise were used for model evaluation. Given that these factors play a major role in PCLC medical device performance assessment, some work is needed to determine the effects of inaccuracy of hardware on model performance assessment. Finally, the results of this research are based on only 16 subjects. This proposed pathway should be further examined when a smaller or larger number of subjects are used for model calibration, which could potentially emphasize the need for comparison methods between candidate models and lead to best practices for computational models based on the subject sample size.

## Conclusion

This research developed tools for model selection and evaluation of first-principle physiological models for use in PCLC medical device assessment. As a case study, we used two physiological models of BV response to fluid perturbation; one developed in our prior work and another expanded from the original model in this research. The models were compared in three aspects of identifiability and interpretability, model calibration, and predictive capability performance. We showed that between the two candidate mathematical models, the refined model performed better, or at least comparable, in all three aspects and thus, it could offer more credibility toward PCLC medical device assessment. The developed pathway in this research can be extended to model selection in other physiological domains.

## Supporting information

S1 File Calibrated parameters for the original model. (PDF)

S2 File Calibrated parameters for the refined model. (PDF)
