# Supplementary material for: Accuracy assessment methods for physiological model selection toward evaluation of closed-loop controlled medical devices
Source: PLoS One. 2021 Apr 30;16(4):e0251001. doi: 10.1371/journal.pone.0251001 (PMC8087034; doi:10.1371/journal.pone.0251001)
Supplement: S1 File — (PDF) [file pone.0251001.s001.pdf]

Calibrated parameters for the original model

	α_u	α_v	K_p	K_i
Subject 1 (LR)	1.4110	1.0903	0.0992	0.0024
Subject 2 (LR)	2.0740	0.4017	0.1252	0.0029
Subject 3 (LR)	2.0707	2.8004	0.0394	0.0018
Subject 4 (LR)	2.07017	0.6142	0.1423	0.0054
Subject 5 (LR)	2.4782	0.6695	0.0926	0.0029
Subject 6 (LR)	1.7065	1.2306	0.0879	0.0011
Subject 7 (LR)	2.2274	2.2000	0.0906	0.0015
Subject 8 (LR)	2.4273	0.7454	0.0577	0.0012
Subject 9 (LR)	0.6923	0.5571	0.0164	0.0012
Subject 10 (LR)	0.6966	0.2657	0.0140	0.0041
Subject 11 (LR)	1.0430	1.1055	0.0867	0.0042
Subject 12 (HEX)	-0.6136	0.3760	0.0395	0.0073
Subject 13 (HEX)	0.2182	1.2473	0.2463	0.0039
Subject 14 (HEX)	-0.0658	1.3999	0.1097	0.0016
Subject 15 (HEX)	-0.1522	1.0387	0.1582	0.0081
Subject 16 (HEX)	-0.2892	0.4852	0.0780	0.0122
