# Supplementary material for: Accuracy assessment methods for physiological model selection toward evaluation of closed-loop controlled medical devices
Source: PLoS One. 2021 Apr 30;16(4):e0251001. doi: 10.1371/journal.pone.0251001 (PMC8087034; doi:10.1371/journal.pone.0251001)
Supplement: S2 File — (PDF) [file pone.0251001.s002.pdf]

Calibrated parameters for the refined model

	A_u	A_v	α_u	α_v	K_p	K_i
Subject 1 (LR)	-0.0459	-0.0106	0.1312	0.6070	1.2230	0.0331
Subject 2 (LR)	-0.0442	-0.0060	0.6551	0.1933	0.0440	0.0230
Subject 3 (LR)	-0.2285	-0.0159	-0.7798	0.8601	0.0986	0.0031
Subject 4 (LR)	-0.0427	-0.0037	0.5526	0.4423	0.1679	0.0154
Subject 5 (LR)	-0.1084	-0.0077	0.2811	0.3941	0.2102	0.0075
Subject 6 (LR)	-0.0035	-2E-08	0.9152	0.9415	0.0940	0.0021
Subject 7 (LR)	-0.0029	-3E-10	1.4288	1.8274	0.0974	0.0024
Subject 8 (LR)	-0.0525	-0.0135	0.3738	0.2282	0.4295	0.0086
Subject 9 (LR)	-0.0216	-0.0020	-0.3121	0.3390	0.0094	0.0061
Subject 10 (LR)	-0.5304	-0.0042	-0.3692	0.3477	0.0772	0.0042
Subject 11 (LR)	-0.6823	-0.0178	-0.7150	0.4248	0.6264	0.0208
Subject 12 (HEX)	-2E-10	-0.0013	-0.5336	0.3394	0.0366	0.0076
Subject 13 (HEX)	-0.0143	-0.0050	-0.0368	0.8950	0.4089	0.0098
Subject 14 (HEX)	-0.0078	-0.0136	0.0472	0.5563	0.2182	0.0131
Subject 15 (HEX)	-3E-08	-0.0040	0.1244	1.0303	0.1624	0.0064
Subject 16 (HEX)	-9E-10	-0.0062	0.0887	0.4015	0.1001	0.0109
